# Prevention and Control of Nosocomial Infections among Hospital Logistic Staff During the COVID-19 Pandemic

**DOI:** 10.1155/2022/5020154

**Published:** 2022-02-12

**Authors:** Zhang Honghui, Li Qingting, Chen Yao, Peng Hua, Geng Chunmi, Long Qing, Guo Jia

**Affiliations:** ^1^Department of Hepatobiliary Surgery, Hunan Provincial People's Hospital: Hunan Normal University First Hospital, Changsha, Hunan, China; ^2^Xiangya School of Nursing, Central South University, Changsha, Hunan, China; ^3^Department of Nursing, Xiangya Hospital of Central South University, Changsha, Hunan, China

## Abstract

Hospital logistics staff comprise a crucial, albeit easily overlooked, group in the prevention and control of hospital nosocomial infections, especially during the COVID-19 pandemic. From the perspective of hospital safety improvement, this paper describes the current situation and challenges in the prevention and control of nosocomial infections among hospital logistics staff. We also provide the following suggestions to improve nosocomial infections: (1) updating the learning system of nosocomial infections, (2) reinforcing the training of infections prevention, (3) properly arranging manpower and resources, and (4) improving the supervision and management system.

## 1. Introduction

Nosocomial infections, also known as hospital-acquired/associated infections, refer to infections occurring within 48 hours of hospital admission, 3 days of discharge, or 30 days of an operation [1, 2]. Nosocomial infections cause global concern with a prevalence as high as 7% in developed countries and 10% in developing countries [3]. In other words, every one in ten patients admitted to a hospital may acquire a nosocomial infection during their stay in developing countries [1–3]. With such a high prevalence, nosocomial infections cause a huge disease burden all over the world, leading to a prolonged hospital stay, increased antimicrobial resistance, higher medical expenses, more medical disputes, long-term disability, and even increased mortality [4]. It is both important and urgent to understand the current situation and risk factors for nosocomial infections in order to guide its further prevention and control.

While medical staff, such as doctors and nurses, have received extensive training on nosocomial infections awareness and prevention, other staff, especially logistics staff, in the hospital received far less attention in the management of nosocomial infections prevention. Hospital logistic staff are a crucial part of the hospital and play essential roles in maintaining the normal operation and management of hospital routine activities. Logistics staff account for more than 10% of the total hospital staff and are highly involved with multiple daily duties including cleaning, sanitizing, collecting and transporting the medical waste, and cleaning contaminated materials, etc. Due to their diverse and extensive daily duties, logistics staff are in close contact with patients, which may expose the patients in a potential risk of nosocomial infections [5–7].

The global pandemic of the COVID-19 further aggravates the severity of nosocomial infections, especially after several outbreaks being reported in China. According to the current policy and guideline on COVID-19 management, all outpatient clinics of a hospital will be closed once a hospital staff was suspected to be infected with COVID-19. Such a strict lockdown policy not only negatively affects the normal operation of hospitals, but more importantly, may cause great inconvenience in patients' visits to hospitals to get timely and proper treatment for their own diseases. Therefore, hospital logistics staff, as a key chain in maintaining hospital normal operation, cannot be ignored in the prevention and control of nosocomial infections. This paper describes the current situation and challenges in the prevention and control of nosocomial infections among hospital logistics staff using a provincial hospital as an example, based on which we further propose several strategies to control nosocomial infections.

## 2. Current Situation and Challenges

### 2.1. Low Education Level and Lack of Professional Knowledge

Hospital logistics staff are characterized by low education levels and have a poor understanding of nosocomial infections; they also lack the professional knowledge in appropriate prevention and control of nosocomial infections [8]. Among the 262 logistics staff employed in the hospital, most have an education level of junior high school or primary school (86.8%), while the remaining 13.2% have completed high school education, indicating a generally low level of education. In addition, these workers have no background in nosocomial infections education or training and do not have the necessary knowledge or awareness on nosocomial infections [9].

### 2.2. Heavy Workload and Low Wages

Hospital logistics staff often undertake multiple tasks such as cleaning, sanitizing, delivering medicines, and maintaining elevator operation, leading to a heavy workload on these staff. However, most logistics staff are hired through an outsourcing company, which usually cuts down the number of workers to reduce labor costs and increase company benefits. As a result, the few limited logistics staff working in the hospital are overloaded with extensive logistics work and are at high risk of burnout. In addition, the wages of logistics staff are very low and do not increase with an increased workload, further discouraging staff's enthusiasm in work. This situation is even worsened by the COVID-19 pandemic that is characterized by its sudden outbreak, destructiveness, and uncertainty, leading to a large number of logistics staff resigning due to excessive fear [10]. All these factors contribute to a serious shortage of logistics staff and a dramatic increase of the workload of the on-the-job front-line logistics staff, leading to poor quality in nosocomial infections [11].

### 2.3. Low Hand Hygiene Compliance and Inadequate Personal Protection

Hospital logistics staff's primary duties include cleaning and disinfecting the hospital environment and medical products for the cleanliness and safety of the hospital. Multiple pathogens may exist in patients' body fluids such as blood, urine, and secretions, as well as medical wastes, object surfaces, and the surrounding air, making it imperative to strictly implement standard protective measures among logistics staff [12]. However, most logistics staff lack the basic knowledge and understanding of the prevention and control of nosocomial infections [12], and without the knowledge of appropriate use of protective equipment [13], thus leading to very low compliance of hand hygiene procedures. A survey has reported that only 16% of logistics staff understood the hand hygiene sign and none mastered the correct hand hygiene procedures [14]. In addition, it has been widely reported that logistics staff have inadequate personal protections. For example, some studies reported that logistics staff were not wearing face masks and gloves at work when they were in contact with body fluids [15, 16]. Other studies reported that protective equipment such as clothing and face masks were not immediately replaced, or even repeatedly used after being contaminated [10, 11, 14, 17].

### 2.4. Lack of Standardized Training and Monitoring

Most of the hospitals only focus on the training and monitoring of nosocomial infection among clinical medical staff such as doctors and nurses but ignore the training and monitoring among logistics staff. There is a lack of comprehensive training requirements and plans on nosocomial infections among newly employed logistics staff [18]. Without appropriate education and training, it may be difficult for logistics staff to realize the importance of nosocomial infections prevention and control and to take effective precautionary measures to reduce and prevent nosocomial infections. Furthermore, the existent problems in nosocomial infections prevention and control may not be corrected in time, thereby further increasing the risk of nosocomial infections among logistics staff. A survey of hospital cleaners showed that 88% of cleaners thought it is necessary to receive standardized training on nosocomial infections prevention and control before entering the ward [17].

## 3. Suggestions for Prevention and Control

### 3.1. Updating the Learning System

The prevention and control measures of nosocomial infections may vary according to the various positions the logistics staff hold in various time periods. This requires targeted training to be implemented among logistics staff based on their requirements for various positions in various time periods [19]. The “Basic Regulations for Nosocomial Infections in Medical Institutions” and “Regulations for Disinfection and Isolation” have listed specific requirements and key points for the prevention and control of nosocomial infections among logistics staff in various positions. For instance, a hospital in Hunan Province has implemented a protocol for infectious diseases transmitted by droplets and/or contact. This protocol has listed specific guidance on the protection standards and equipment configuration for logistics staff in different positions and different regions during the COVID-19 pandemic.  Table 1 shows the details of the protocol that include the “Guidelines for Disinfection in Public Areas,” “Management List of Disinfection and Isolation Items for Key Departments,” and “Specific Rules for Disinfection and Isolation in Nonquarantine Areas.”

### 3.2. Strengthening the Training System

Once the learning system on nosocomial infections is updated, logistics staff will be immediately organized to receive standardized training. The training is mainly led by the head nurse and the nurses in charge of nosocomial infections and is composed of two major parts: theoretical training and skills training. Theoretical training includes the following parts: (1) knowledge on infectious diseases, such as the etiology, transmission routes, and basic protective measures of COVID-19; (2) knowledge on nosocomial infections, such as various disinfection requirements in various regions, specimen transfer, and medical waste disposal; (3) knowledge on self-protection, such as face-masks wearing, hand hygiene procedures, temperature monitoring, and reporting. Skills training includes appropriate hand hygiene procedures, face-masks wearing, and protective clothing wearing and takeoff. Considering the general low literacy level of the logistics staff, the training will take diverse forms such as live demonstrations and video shows to improve the training effect [20]. After appropriate training, the hand hygiene compliance of the logistics staff in the hospital has increased from 36% to 95%.

### 3.3. Properly Arranging Manpower and Resources

The COVID-19 pandemic has led to a widespread shortage of staff and medical resources at various positions in all hospitals. Hospitals should actively mobilize resources from all over the country to meet various position requirements and ensure normal resources supply. The logistics management department of the hospital should negotiate with the outsourcing company to protect the rights and well-being of logistics staff including increasing the number of staff workers, decreasing their workloads, and enhancing effective communications. In addition, the hospital should also provide encouragement and support to alleviate psychological distress such as panic, depression, and anxiety caused by work overload and fear of infections. Negative emotions among logistics staff during the COVID-19 may lead to the ignorance of and inadequate implementation of nosocomial infections prevention and control measures, thus increasing the risk of nosocomial infections. In addition, the hospital should distribute protective resources based on the actual needs of each post in each department and strengthen the management of these protective resources. The logistics staff should be instructed to take appropriate protective measures according to their exposure risk while avoiding resources waster caused by overprotection [6].

### 3.4. Improving the Supervision and Management System

Hospitals should attach more importance to their logistics staff and understand the essential roles they play in the prevention and control of nosocomial infections. Hospitals should strengthen cooperation with outsourcing companies to establish a complete, orderly, and feasible management model that connects the hospital logistics management department, hospital nosocomial infections control department, hospital clinical departments, and outsourcing companies. This multiparty comanagement model establishes a joint inspection team to conduct on-site inspections and enables each department to implement its own function, as well as strengthens communications among departments [21]. In addition, hospitals should establish objective evaluation standards and improve the evaluation system to strengthen the supervision and management of logistics staff. The logistics management personnel should conduct regular and irregular random inspections on the logistics staff to assess their knowledge and implementation of disinfection and isolation. During the COVID-19 pandemic, the hospital is encouraged to adopt a dual-post system, where two logistics staff are arranged for each post to assist and monitor each other at work. The hospital should also encourage patients and other medical staff to monitor the logistics staff's work so as to establish a multiparty monitoring mechanism. The fixed-post system should be replaced by a rotation system to ensure that the logistics staff can quickly master their job skills and reduce the occurrence of errors. The rotation time of logistics staff should be determined based on their specific duties and a reasonable rewarding system should be in place to incentivize logistics staff's work enthusiasm.

## 4. Summary

Since the outbreak of COVID-19 in 2020, the National Epidemic Prevention and Control Department has issued multiple policy documents on infections prevention, emphasizing the importance of nosocomial infections prevention and control. Whether for daily medical management or for the fight against the COVID-19 pandemic, the prevention and control of nosocomial infections in medical institutions have always been a priority. Logistics staff are a crucial part of the hospital and their important roles in maintaining the hospital's normal operation can never be ignored. Logistics staff are characterized by heavy workloads, high personnel mobility, and low education levels, making it difficult to implement standard protection and constituting a weak link in the prevention and control of nosocomial infections. In recent years, there have been reported cases of nosocomial infections among logistics staff in the fight against major infectious diseases. Based on the past experiences and challenges in the prevention and control of nosocomial infections, the establishment of a comprehensive training and monitoring system for nosocomial infection prevention and control among logistics staff carries significant implications and may be extended to other countries.

## Figures and Tables

**Table 1:** List of personal protection standards and supplies for workers and staff in different regions and jobs.

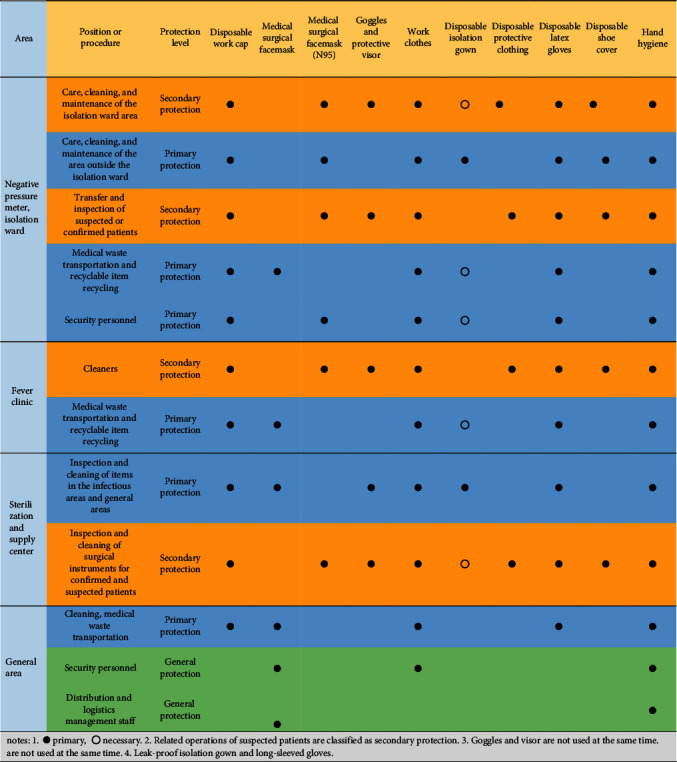

## Data Availability

The data that support the findings of this study are available from the corresponding author upon request.
